# Comprehensive GC-MS Measurement of Amino Acids, Metabolites, and Malondialdehyde in Metformin-Associated Lactic Acidosis at Admission and during Renal Replacement Treatment

**DOI:** 10.3390/jcm13133692

**Published:** 2024-06-25

**Authors:** Rene A. Posma, Stephan J. L. Bakker, Maarten W. Nijsten, Daan J. Touw, Dimitrios Tsikas

**Affiliations:** 1 Department of Critical Care, University of Groningen, University Medical Center Groningen, 9713 GZ Groningen, The Netherlands; r.a.posma@umcg.nl (R.A.P.); m.w.n.nijsten@umcg.nl (M.W.N.); 2 Division of Nephrology, Department of Internal Medicine, University of Groningen, University Medical Center Groningen, 9713 GZ Groningen, The Netherlands; s.j.l.bakker@umcg.nl; 3 Department of Clinical Pharmacy and Pharmacology, University of Groningen, University Medical Center Groningen, 9713 GZ Groningen, The Netherlands; d.j.touw@umcg.nl; 4 Institute of Toxicology, Core Unit Proteomics, Hannover Medical School, 30623 Hannover, Germany

**Keywords:** amino acids, GC-MS, intoxication, MALA, oxidative stress, renal replacement therapy

## Abstract

Metformin is the most widely used drug in type 2 diabetes. Regular metformin use has been associated with changes in concentrations of amino acids. In the present study, we used valid stable-isotope labeled GC-MS methods to measure amino acids and metabolites, including creatinine as well as malondialdehyde (MDA), as an oxidative stress biomarker in plasma, urine, and dialysate samples in a patient at admission to the intensive care unit and during renal replacement treatment because of metformin-associated lactic acidosis (MALA, 21 mM lactate, 175 µM metformin). GC-MS revealed lower concentrations of amino acids in plasma, normal concentrations of the nitric oxide (NO) metabolites nitrite and nitrate, and normal concentrations of MDA. Renal tubular reabsorption rates were altered on admission. The patient received renal replacement therapy over 50 to 70 h of normalized plasma amino acid concentrations and their tubular reabsorption, as well as the tubular reabsorption of nitrite and nitrate. This study indicates that GC-MS is a versatile analytical tool to measure different classes of physiological inorganic and organic substances in complex biological samples in clinical settings such as MALA.

## 1. Introduction

Metformin (*N*,*N*-dimethylbiguanide) is the most commonly prescribed oral antihyperglycemic drug to treat type 2 diabetes [1]. Metformin is eliminated in urine in its unchanged form via renal secretion [2]. Patients with metformin intoxication can develop metformin-associated lactic acidosis (MALA), a life-threatening condition. MALA is defined as a plasma pH < 7.35, a plasma lactate concentration >5 mM, and a metformin concentration >5 mg/L [3]. The pathophysiology of MALA is characterized by reduced lactate clearance due to impaired gluconeogenesis combined with inhibition of systemic mitochondrial respiration, causing an increase in anaerobic metabolism [4,5,6,7]. Patients with MALA are treated in the intensive care unit (ICU), requiring comprehensive supportive care in combination with renal replacement therapy (RRT), such as intermittent hemodialysis (HD) or continuous RRT [8].

Metformin use has been associated with changes in concentrations of amino acids and their metabolites, including homoarginine [9,10]. The effects of metformin-induced post-translational modifications (PTM) at toxic concentrations are scarce [11,12]. In urine of a patient with MALA, we have previously found an accumulation of hydroxy-proline (OH-Pro) [13]. The aim of the present study was to determine the status of other amino acids, including arginine and some of its metabolites, and of oxidative stress in the MALA patient at ICU admission and during RRT. Free L-arginine (Arg) is the physiological substrate of nitric oxide synthase (NOS) isoforms [14]. Of particular interest were the major metabolites and measures of nitric oxide (NO), namely nitrate and nitrite. Post-translational dimethylation of Arg residues in proteins leads to symmetric dimethylarginine (SDMA) and asymmetric dimethylarginine (ADMA), which are endogenous inhibitors of NOS activity [14]. As dimethyl amine (DMA) is the major urinary metabolite of ADMA [15], it was of interest to investigate potential effects of MALA on this ADMA metabolite. Eventually, malondialdehyde (MDA) was measured as a biomarker of oxidative stress [16] in the MALA patient.

Mass spectrometry coupled to gas chromatography (GC-MS) is a widely used instrumental technique for the reliable quantitative determination of numerous physiological compounds, drugs, and their metabolites in biological samples such as plasma and urine. In the present work, we used previously described validated GC-MS methods for the measurement of the above-mentioned analytes [17,18,19,20,21,22,23,24]. These methods include use of suitable derivatization techniques for the analytes and their stable-isotope labeled analogs (^2^H, ^15^N), which serve as internal standards. All GC-MS analyses in this study were performed in the negative-ion chemical ionization (NICI) mode and by using selected-ion monitoring (SIM) of analyte-specific ions.

## 2. Materials and Methods

### 2.1. Patient

The case report on metformin intoxication and RRT treatment has been described elsewhere in detail [13] and is briefly summarized below.

The 70-year old female patient (bodyweight, 84 kg) was oliguric, and the plasma creatinine concentration was 606 µM, indicating acute renal failure. The patient was transferred to our hospital to start RRT. At ICU admission, the blood metformin concentration was 175 µM. During hemodialysis, the metformin plasma concentration rapidly decreased, and the acidosis improved, with an increase in pH from 7.26 to 7.40 in approximately 2 h. Likewise, lactate concentration decreased from 21 mM to <2 mM within 12 h. After hemodialysis (HD) was stopped, continuous veno-venous hemodiafiltration (CVVHDF) and, subsequently, continuous veno-venous hemofiltration (CVVH) was applied. Heparin was used as an anticoagulant during CVVHDF, whereas citrate was used during CVVH. After 12 h of ICU admission, the metformin concentration decreased to 39 µM, which is generally considered to be non-toxic [25]. Heparinized plasma, urine, and dialysate samples were centrifuged and stored at −80 °C. Written informed consent was obtained to collect residual material and for publication of this case report. Ethical approval was given by the institutional review board (METc 2014-552).

### 2.2. GC-MS Analyses in Plasma, Urine, and Dialysate Samples

Metformin, amino acids, nitrate, nitrite, and MDA were analyzed by GC-MS in plasma, urine, and dialysate (effluent) samples after suitable derivatization, as described previously [17,18,19,20,21,22,23,24]. All amino acids were measured simultaneously as methyl ester pentafluoropropionyl derivatives. Nitrate, nitrite, creatinine, and MDA were measured simultaneously as pentafluorobenzyl derivatives. DMA was analyzed after extractive derivatization as a pentafluorobenzamide derivative. In all analyses, commercially available and de novo synthesized stable-isotope labelled analogs were used as internal standards for each analyte.

GC-MS analyses were performed on a single quadrupole mass spectrometer model ISQ, equipped with a Trace 1210 series gas chromatograph, and an AS1310 autosampler (ThermoFisher, Dreieich, Germany). A fused-silica capillary column Optima 17 (15 m length, 0.25 mm I.D., 0.25 µm film thickness) was used (Macherey-Nagel, Düren, Germany). Methane was used as the reagent gas (flow rate of 2.4 mL/min) for NICI. Quantitative analyses were performed in the SIM mode. The GC-MS method for amino acids measures the sum of citrulline (Cit) and ornithine (Orn), asparagine (Asn) and aspartate (Asp), glutamine (Gln) and glutamate (Glu), leucine (Leu) and isoleucine (Ile). The concentrations of these amino acids are reported as Orn, Asp, Glu, and Leu, respectively. Analyte concentrations in urine were corrected for urinary creatinine excretion and are reported as µmol analyte per mmol creatinine. Creatinine in plasma and urine was also analyzed by GC-MS, as described elsewhere [24]. All analyses were performed at the same time in one laboratory by a single skillful person.

### 2.3. Calculations and Statistical Analyses

Fractional excretion (FE, %) values were calculated for all analytes by dividing the concentration ratio of creatinine (Crea) in plasma (P) and urine (U), i.e., [Crea]_P_/[Crea]_U_, by the concentration ratio of an analyte (A) in plasma and urine, i.e., [A]_P_/[A]_U_, measured at a certain time point, and by multiplying the result by 100 (see Formula (1)). Tubular reabsorption values (T, %) of analytes were calculated by subtracting the FE values from 100 (see Formula (2)).

FE (%) = ([Crea]_P_/[Crea]_U_)/([A]_P_/[A]_U_) × 100% (1)

T (%) = 100 − FE (%)(2)

Renal clearance was calculated by dividing the elimination rate in urine from the previous time-point, at which urine was collected to the current time-point by the corresponding plasma concentration. The instantaneous plasma clearance by dialysis was calculated by dividing the product of dialysate flow and concentration in the dialysate (i.e., elimination rate) by the plasma concentration. Because the blood samples were not simultaneously obtained together with the effluent samples, the concentration in plasma was linearly interpolated using data from four time-points with the Trend function in Excel (Microsoft, Redmond, WA, USA). Calculating plasma clearance by dialysis using the AV method was not possible because no simultaneous inflow and outflow samples were obtained. To present the average clearance of each compound during CVVHDF and CVVH, respectively, the time-weighted mean clearance was calculated, which is more representative of the true clearance than the arithmetic mean, as it assumes a linear trend between each individual measurement [26].

Data analyses were performed with GraphPad Prism version 7 (GraphPad Software, San Diego, CA, USA) and R version 4.3.2 (R Foundation for Statistical Computing, Vienna, Austria).

## 3. Results and Discussion

**Figure 1:**
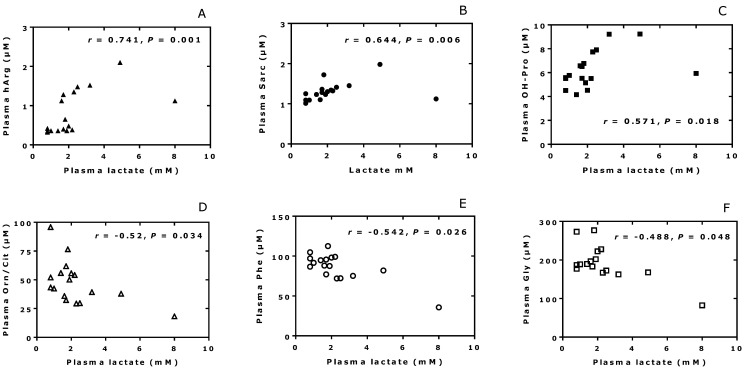
Correlations (after Spearman) between the plasma concentrations of the indicated amino acids and lactate measured in the MALA patient.

**Figure 2:**
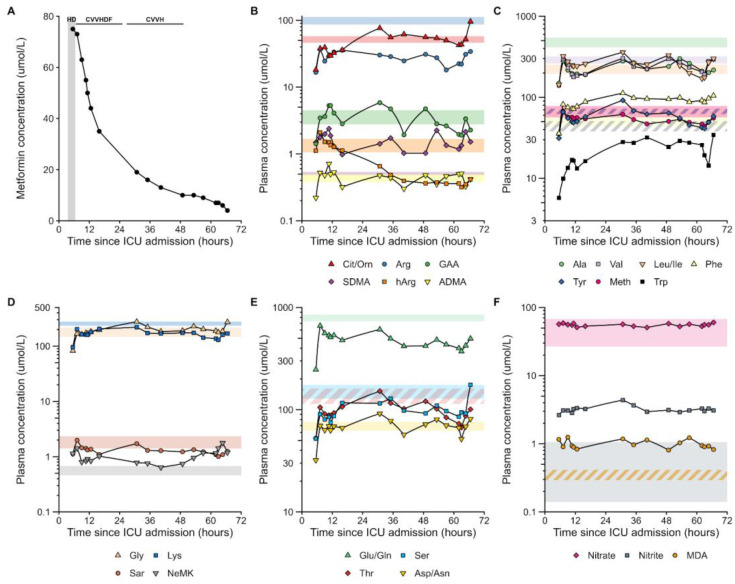
Time course of the plasma concentrations of metformin (**A**), the indicated amino acids (**B**–**E**), and nitrate, nitrite, and malondialdehyde (MDA) (**F**) during the renal replacement treatment (RRT) of the patient in the ICU since admission. HD, hemodialysis; CVVHDF, continuous veno-venous hemodiafiltration; CVVH, continuous veno-venous hemofiltration (CVVH). Shaded areas are reference values (interquartile range) obtained from healthy volunteers.

The concentrations of the analytes measured in the samples of the study are presented in Table 1 and Table 2 and in Figure 1 and Figure 2. For the sake of clarity and better readability, the results are presented separately for the amino acids and the other analytes. For comparison of analyte concentrations measured in the patient’s samples, we used concentrations of the analytes as measured in studies on healthy subjects [17,18,19,20,21,22,23,24,27,28,29,30,31,32,33,34,35,36,37]. These data are summarized in Table 3. The reference interval for plasma creatinine was reported to be 53–115 µM [36].

In general, the plasma concentrations of the analyzed amino acids were in the lower range of reference intervals. The concentrations of nitrate, nitrite, and MDA measured in plasma and urine were within normal ranges. These observations suggest rather unaltered amino acid, NO homeostasis, and oxidative stress. The results of the study are discussed in detail as follows below for plasma, urine, and effluent (dialysate).

At admission, the plasma Arg concentration was lower compared to healthy humans (Table 2 and Table 3). The plasma concentration of the Arg metabolite ADMA was also lower than in healthy adults. The SDMA plasma concentration was higher, probably due to decreased urinary excretion of this Arg metabolite, which is eliminated primarily unchanged by glomerular filtration. The plasma concentration of guanidinoacetate (GAA), an Arg metabolite from the arginine:glycine amidinotransferase (AGAT) pathway and the precursor of creatine, remained within normal ranges. The plasma concentration of hArg, another Arg metabolite from the AGAT pathway, was normal at ICU admission but decreased over time. The plasma concentrations of N^ε^-methyl lysine (N^ε^MK), a PTM metabolite of lysine (Lys) residues in proteins, were considerably higher than in healthy subjects, indicating a higher monomethylation extent of Lys in the MALA patient. At admission, the plasma concentrations of nitrate, nitrite, and MDA were within normal ranges (Table 1 and Table 3). Thus, NO synthesis and oxidative stress were not elevated in the MALA patient and did not change with renal replacement treatment.

Except for hydroxy-proline (−0.9-fold) and homoarginine (−2.7-fold), at the end of the renal replacement treatment, the plasma amino acid concentrations were up to 6-fold higher compared to those at admission to the ICU (Table 3). The highest increase was observed for tryptophan (6-fold).

We tested for potential correlations between the plasma concentrations of lactate and other analytes. The plasma lactate concentration values correlated positively with the plasma concentrations of homoarginine (hArg; *r* = 0.741, *p* = 0.0007), sarcosine (Sar; *r* = 0.644, *p* = 0.005), and hydroxy-proline (OH-Pro; *r* = 0.5707, *p* = 0.0184), yet not of proline [13] (Figure 1). Lactate has been shown to inhibit proline oxidase activity [35]. The correlation was negative for phenylalanine (Phe; *r* = −0.521, *p* = 0.025), ornithine (Orn; *r* = −0.520, *p* = 0.032), and glycine (Gly; *r* = −0.488, *p* = 0.047) (Figure 1). The plasma lactate concentrations did not correlate with the plasma concentrations of nitrite, nitrate, or MDA. These associations could result from effects of lactate of enzyme activities involved in the metabolism of amino acids [35], but this remains to be investigated in other cases of MALA. Lack of correlation between plasma concentrations of nitrite, nitrate, or MDA and lactate was also observed in healthy young men [34].

The urinary excretion rate of ADMA was lower than in healthy adults. The excretion rate of the ADMA metabolite DMA was higher than in healthy adults. This observation suggests increased metabolism of ADMA to DMA, presumably due to elevated activity of dimethylarginine dimethylaminohydrolase (DDAH) that hydrolyses ADMA to DMA and citrulline.

**Figure 3:**
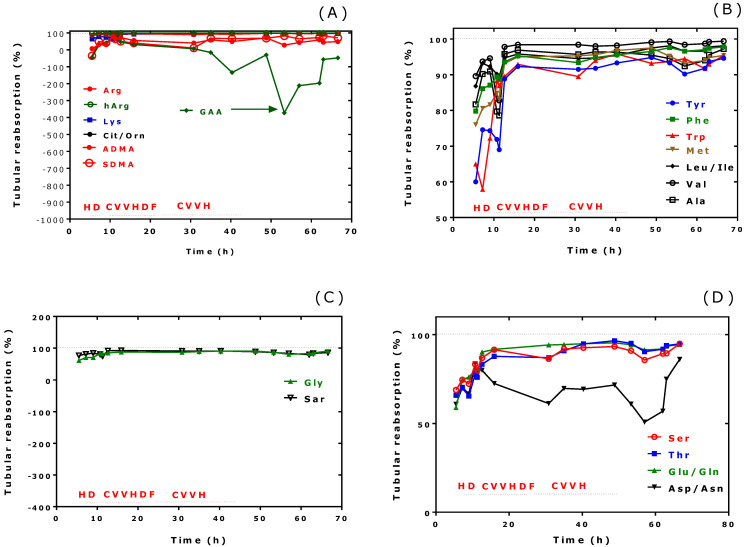

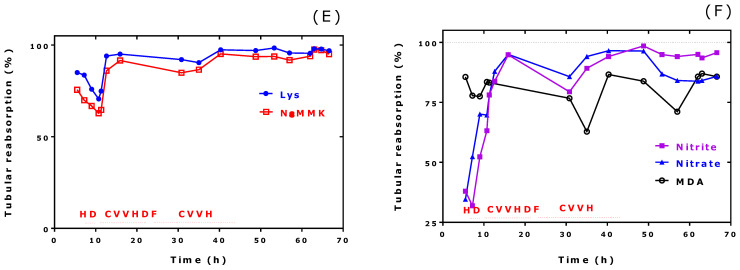
Time course of the calculated tubular reabsorption rates of the indicated amino acids, nitrate, nitrite, and malondialdehyde (MDA) during the renal replacement treatment (RRT) of the patient in the ICU since admission. HD, hemodialysis; CVVHDF, continuous veno-venous hemodiafiltration; CVVH, continuous veno-venous hemofiltration (CVVH).

The tubular reabsorption values of most amino acids (Figure 3A–E) and of nitrite and nitrate (Figure 3F) were low and increased within the first hours of extracorporeal treatment to reach normal values. The tubular reabsorption values of GAA and Asn/Asp differed from those of the other amino acids. The negative tubular reabsorption values seen for GAA (Figure 3A) and Asn/Asp (Figure 3D) are indicative of renal secretion of these amino acids. During ICU admission, urinary Lys and N^ε^MK excretion decreased temporarily (Figure 3E), suggesting improved tubular reabsorption over time. The low tubular reabsorption values of nitrate and nitrite within the first hours of treatment indicate altered renal management of these NO metabolites in the MALA patient.

## 4. Conclusions

Stable-isotope dilution GC-MS allows quantitative analysis of structurally different physiological inorganic (nitrite, nitrate), organic hydrophilic (amino acids and metabolites), and lipophilic (MDA) substances, as well as drugs (metformin) in complex biological samples such as plasma and urine. Our present and previous [13] study suggest that metformin-intoxication is not associated with greatly altered amino acid homeostasis, except for an elevated PTM of proline by prolyl-hydroxylation. This PTM seems to be specific for the MALA patient since other PTM metabolites, including those of Arg (i.e., ADMA, SDMA) and Lys (i.e., N^ε^MK), were not elevated at admission and did not change remarkably during the renal replacement treatment. That the plasma MDA concentration was not elevated in the MALA patient at admission and did not change during the renal replacement therapy suggests that oxidative stress is not associated with MALA. Extracorporeal renal replacement therapy is efficient in MALA, both with respect to metformin elimination and normalization of the renal management of solutes. In this case report, we have analyzed a single MALA patient. This is a study limitation. Confirmation of the observations in this patient is warranted by analyzing plasma, urine, and dialysate samples from additional metformin-intoxicated patients.

## Figures and Tables

**Table 1:** (**a**) Concentrations of metformin, creatinine, and metabolites (all in µM) in plasma samples of the patient during (time in h) RTR. (**b**) Concentrations of metformin (mM), creatinine (mM), and metabolites (all in µM) in urine samples of the patient during (time in h) RTR. (**c**) Concentrations of metformin and metabolites (all in µM) in effluent samples of the patient during (time in h) RTR.

**(a)**									
**Time**	**Metformin**	**Creatinine**	**Nitrate**	**Nitrite**	**MDA**		**GAA**	**ADMA**	**SDMA**
*5.5*	*75*	*388*	*57*	*2.64*	*1.16*		*1.42*	*0.22*	*1.48*
7.2	73	385	59	3.09	0.90		3.46	0.53	1.76
9.0	63	350	56	3.07	1.25		3.63	0.48	1.98
10.7	55	297	55	2.83	0.93		5.30	0.71	2.38
11.3	50	307	58	3.24	0.89		5.31	0.50	1.88
12.6	44	267	51	3.34	0.83		4.09	0.53	1.30
15.9	35	202	53	3.25	0.07		2.83	0.32	0.98
30.8	19	183	57	4.38	1.18		5.85	0.48	1.42
35.0	16	167	53	3.66	0.96		4.72	0.44	1.73
40.3	13	145	51	2.96	1.14		1.93	0.30	1.03
48.8	10	135	58	3.12	0.81		4.74	0.48	1.03
53.3	10	190	53	2.92	1.03		2.82	0.35	2.25
57.0	9	219	57	3.08	1.23		2.62	0.46	1.34
62.0	7	250	53	3.29	0.94		1.96	0.50	1.18
63.0	7	226	56	3.02	0.90		1.91	0.51	1.34
64.7	6	234	56	3.29	0.92		3.35	0.33	2.15
66.6	4	230	60	3.08	0.82		2.28	0.42	1.52
**(b)**									
**Time**	**Metformin**	**Creatinine**	**DMA**	**Nitrate**	**Nitrite**	**MDA**	**GAA**	**ADMA**	**SDMA**
*5.5*	*1.46*	*2.5*	*126*	*239*	*10.5*	*1.08*	*13.2*	*1.32*	*12.9*
7.2	1.17	1.88	84	137	10.3	0.98	12.2	1.8	4.3
9.0	1.44	2.23	124	107	9.3	1.79	13.6	2.14	7.2
10.7	1.23	2.11	102	119	7.4	1.09	11.0	1.07	5.0
11.3	1.25	2.19	105	91	5.1	1.08	11.8	1.23	4.8
12.6	2.58	4.84	213	112	9.8	15.07	39.1	2.68	11.8
15.9	3.37	6.14	279	81	5.1	19.52	59.6	4.39	17.8
30.8	3.5	4.88	321	216	24.1	7.31	146.1	7.65	33.7
35.0	2.87	4.49	231	84	10.6	9.66	147.8	4.93	14.8
40.3	4.02	6.29	367	78	7.5	6.64	196.4	6.71	15.1
46.5	5.11	6.39	423	83	5.4	6.88	212.8	8.45	15.6
48.8	3.28	5.43	360	84	1.9	5.28	248.2	5.78	13.3
50.8	2.04	3.33	230	86	1.8	3.53	198.4	4.15	6.1
53.3	1.17	2.56	191	93	2.0	10.81	179.7	3.42	5.4
57.0	0.95	2.61	148	108	2.2	4.22	97.5	3.17	5.7
62.0	0.68	3.17	153	127	2.1	1.81	79.7	3	5.1
63.0	0.64	3.6	157	137	3.3	1.85	81.1	2.79	5.7
64.7	0.6	3.85	162	141	3.1	1.7	59.8	3.15	6.0
66.6	0.5	4.04	233	150	2.3	2.07	59.4	3.85	7.8
**(c)**									
**Time**	**Metformin**	**DMA**	**Nitrate**	**Nitrite**	**MDA**	**Creatinine**	**GAA**	**ADMA**	**SDMA**
*5.0*	*28.6*	*3.41*	*31.1*	*9.49*	*0.19*	*155*	*1.67*	*0.16*	*0.54*
8.8	80.9	10.5	32.2	2.70	0.27	344	2.00	0.40	1.52
11.0	69.6	9.09	25.9	4.48	0.25	427	2.49	0.32	1.22
12.0	60.7	7.73	29.0	4.13	0.29	384	2.94	0.31	1.39
13.0	54.9	7.80	24.3	4.12	0.27	423	3.97	0.32	1.31
15.3	45.2	7.92	27.5	3.89	0.23	415	3.04	0.31	1.36
17.6	37.1	6.86	25.3	3.92	0.26	344	2.38	0.28	0.93
19.5	32.5	6.96	21.4	4.10	0.22	256	3.85	0.31	1.09
20.8	29.7	6.14	24.7	4.08	0.20	256	2.36	0.32	1.01
24.3	23.3	4.50	21.7	3.62	0.23	188	4.32	0.36	0.76
29.1	20.6	7.16	29.6	3.29	0.21	190	6.12	0.33	1.30
30.5	18.2	5.68	28.3	2.97	0.23	286	5.90	0.30	1.16
33.4	16.2	7.81	24.4	2.93	0.20	245	8.83	0.33	1.27
35.0	15.6	6.11	32.1	5.25	0.22	267	7.48	0.25	1.43
40.3	12.1	6.93	24.3	4.17	0.48	259	3.44	0.28	1.07
42.6	11.9	5.15	24.5	3.82	0.23	252	6.38	0.35	0.94
46.5	10.0	5.54	23.5	3.94	0.19	224	10.73	0.25	1.37
48.9	9.6	5.83	23.8	4.75	0.24	220	5.25	0.30	1.84
50.0	18.0	5.95	25.4	2.67	0.24	277	5.73	0.39	1.58

**Table 2:** (**a**) Concentrations of amino acids (all in µM) in plasma samples of the patient during (time in h) the RTR. (**b**) Concentrations of amino acids (all in µM) in urine samples of the patient during (time in h) RTR. (**c**) Concentrations of amino acids (all in µM) in effluent samples of the patient during (time in h) RTR.

**(a)**																	
**Time**	**Ala**	**Thr**	**Gly**	**Val**	**Ser**	**Sar**	**Leu**	**Asp**	**Gln**	**Met**	**Orn**	**Phe**	**Tyr**	**Lys**	**Arg**	**hArg**	**Trp**
*5.5*	*149*	*53*	*82*	*141*	*52*	*1.12*	*143*	*32*	*247*	*35*	*18*	*36*	*31*	*96*	*17*	*1.12*	*5.7*
7.2	281	106	168	296	90	1.98	322	70	662	67	38	82	66	203	37	2.1	9.9
9.0	217	91	163	231	80	1.45	276	63	560	57	39	75	55	163	25	1.52	13.5
10.7	197	88	173	191	85	1.41	249	69	533	57	30	72	50	162	29	1.48	16.9
11.3	190	87	167	178	75	1.32	243	63	512	55	29	72	49	160	30	1.35	16.5
12.6	194	93	183	185	87	1.36	243	69	534	56	32	77	50	180	34	1.28	13.2
15.9	190	107	197	192	117	1.1	259	66	478	54	36	88	57	205	36	1.12	16.3
30.8	280	152	277	303	116	1.72	359	92	610	62	77	113	92	220	30	0.65	28.0
35.0	268	116	222	240	128	1.3	263	78	496	53	56	98	68	173	29	0.48	27.4
40.3	223	102	184	224	98	1.28	254	57	419	47	62	96	62	169	25	0.40	31.9
48.8	240	121	190	298	92	1.23	330	72	420	50	56	95	64	174	31	0.36	24.3
53.3	301	102	228	271	110	1.34	246	80	483	54	54	99	55	174	28	0.38	29.0
57.0	263	84	202	234	97	1.23	199	70	433	49	50	88	46	143	18	0.36	27.5
62.0	211	73	189	206	86	1.09	170	67	399	47	42	92	42	137	22	0.36	25.8
63.0	178	67	177	190	94	1.01	176	52	373	44	43	87	41	130	22	0.32	19.3
64.7	202	87	187	280	92	1.09	269	69	423	50	52	97	49	160	31	0.35	14.3
66.6	217	101	273	296	175	1.25	301	81	496	55	96	105	58	169	34	0.42	34.3
**(b)**																	
**Time**	**Ala**	**Thr**	**Gly**	**Val**	**Ser**	**Sar**	**Leu**	**Asp**	**Gln**	**Met**	**Orn**	**Phe**	**Tyr**	**Lys**	**Arg**	**hArg**	**Trp**
*5.5*	*175*	*116*	*206*	*95*	*105*	*1.7*	*122*	*81*	*652*	*54.5*	*16.1*	*46.4*	*81*	*224*	*14.9*	*0.27*	*12.9*
7.2	135	155	246	91	112	1.8	107	101	823	63.8	21.0	55.7	82	226	13.9	0.24	20.4
9.0	126	200	309	80	141	1.5	136	134	850	66.6	20.7	61.7	90	252	15.2	0.22	23.8
10.7	284	139	199	227	101	2.0	174	81	762	61.6	24.3	54.2	99	365	14.0	0.52	14.1
11.3	290	150	217	219	108	2.4	182	88	847	66.4	25.5	58.0	108	380	15.9	0.53	15.5
12.6	147	281	506	74	206	2.0	234	250	953	69.2	23.4	88.9	102	282	30.5	0.23	25.1
15.9	179	397	745	94	302	2.5	332	553	1202	83.1	37.6	125.9	135	421	46.8	0.27	35.2
30.8	320	524	985	132	420	4.2	530	946	947	77.5	64.0	198.9	208	480	85.6	0.22	78.6
35.0	258	283	659	129	279	3.3	376	631	745	62.6	38.0	136.5	150	293	57.2	0.18	43.9
40.3	361	233	740	172	315	5.0	507	761	928	65.7	39.4	185.0	179	248	87.5	0.19	57.8
46.5	515	194	854	137	293	8.0	539	939	932	63.1	65.2	176.8	169	235	66.2	0.2	71.4
48.8	432	165	724	113	248	5.6	338	820	784	51.3	46.7	135.1	135	167	41.8	0.13	66.6
50.8	277	152	500	84	206	3.1	204	579	572	43.4	31.3	77.5	91	143	38.8	0.1	44.0
53.3	223	67	405	26	134	2.5	63	421	374	34.8	20.1	32.0	50	35	9.3	0.08	24.7
57.0	237	96	469	43	165	2.4	81	412	444	39.9	15.5	35.6	53	56	11.4	0.08	17.9
62.0	148	77	429	35	142	2.9	84	312	425	37.3	14.7	37.0	48	70	12.2	0.07	22.5
63.0	137	82	437	36	149	2.8	91	264	421	39.6	13.3	39.2	48	75	14.6	0.07	15.8
64.7	118	69	407	31	127	3.0	78	164	427	40.1	14.0	39.0	46	81	12.4	0.07	23.3
66.6	107	93	439	34	158	3.1	102	199	479	44.5	20.0	40.6	55	118	13.6	0.09	27.7
**(c)**																	
**Time**	**Ala**	**Thr**	**Gly**	**Val**	**Ser**	**Sar**	**Leu**	**Asp**	**Gln**	**Met**	**Orn**	**Phe**	**Tyr**	**Lys**	**Arg**	**hArg**	**Trp**
*5.0*	*71*	*17*	*28*	*66*	*11.8*	*0.58*	*55*	*9.2*	*104*	*15.3*	*4.7*	*14.1*	*13.3*	*31*	*7.4*	*0.59*	*2.1*
8.8	203	77	115	238	43.7	2.68	242	40.2	541	38.9	21.3	67.0	58.4	138	38.5	1.71	10.4
11.0	140	64	111	166	35.5	1.37	161	35.1	432	35.2	16.2	56.6	40.8	103	28.5	1.36	6.5
12.0	125	60	109	137	34.5	1.45	139	33.2	421	33.7	15.0	52.7	38.3	108	27.0	1.20	6.8
13.0	135	72	135	153	40.4	1.45	166	41.9	482	36.8	18.1	63.5	42.8	133	33.0	1.21	7.7
15.3	156	88	157	160	51.5	2.02	196	49.2	486	39.0	22.1	71.4	50.5	148	43.0	1.22	10.0
17.6	145	90	152	154	53.0	1.35	192	47.8	426	35.8	21.0	72.0	50.4	148	43.9	0.98	9.7
19.5	181	106	158	179	60.6	1.19	223	52.4	395	34.3	25.4	79.8	60.5	163	50.7	0.84	11.4
20.8	158	125	143	191	65.9	1.58	240	57.0	418	35.1	26.9	83.8	70.8	181	54.3	0.80	12.3
24.3	207	129	170	204	68.3	1.25	249	58.2	375	34.4	28.5	84.4	76.7	175	60.3	0.70	13.2
29.1	205	121	165	210	61.9	1.75	240	52.9	371	34.6	33.2	83.5	77.4	141	46.8	0.64	14.9
30.5	182	123	168	220	62.4	1.88	259	52.4	369	35.6	35.7	85.6	73.0	140	48.9	0.58	15.1
33.4	208	112	156	221	58.4	1.91	227	52.2	358	34.9	34.1	84.1	65.7	121	41.3	0.48	13.6
35.0	187	96	140	181	51.1	1.69	180	45.6	317	31.6	28.3	77.8	54.4	107	34.6	0.46	11.8
40.3	153	83	124	177	46.1	1.28	173	36.3	251	28.0	27.8	74.6	46.6	105	32.6	0.33	11.2
42.6	195	107	139	250	51.1	1.64	246	47.3	318	32.8	47.4	91.6	65.0	130	35.3	0.40	14.3
46.5	180	95	128	215	45.1	1.44	204	46.1	291	29.9	41.8	85.1	56.9	109	28.7	0.33	14.5
48.9	157	101	128	223	52.1	1.13	257	48.8	295	33.7	37.6	81.5	51.4	116	40.1	0.36	17.3
50.0	198	122	162	234	63.9	1.66	267	52.9	367	37.9	36.3	89.5	71.5	136	49.0	0.56	14.2

**Table 3:** Reported plasma/serum concentrations (range, µM) in healthy humans for the analytes measured in the present study at admission to the ICU (5.5 h) until the end (66 h).

**Analyte**	**Men** [34]	**Women** [33]	**MALA Patient** **5.5 h–66 h**	**Fold Change**	**Refs.**
Creatinine	82–101	n.m.	−230	−0.6	[36]
Malondialdehyde	0.31–0.46	n.m.	1.16–0.82	−0.7	[16]
Metformin	n.a.	n.a.	−4	−19.0	n.a.
Nitrate	38–55	n.m.	57–60	+1.0	[14]
Nitrite	1.9–2.6	n.m.	2.64–3.08	+1.2	[14]
**Amino acids**					
ADMA	0.187–0.534	n.m.	0.220–0.420	+1.9	[30]
Alanine	254–392	203–443	149–217	+1.5	[33]
Arginine	50–90	50–119	17–34	+2.0	[33]
Asparagine	48–94	33–59	32–81	+2.5	[33]
Citrulline	34–48	18–42		+2.3	[33]
Glutamine	449–654	414–669 12–57	247–496	+2.0	[33]
Glycine	147–248	149–397	82.0–273	+3.3	[33]
Guanidinoacetate	1.77–4.57	n.m.	1.42–2.28	+0.9	none
Homoarginine	0.66–1.74	n.m.	1.12–0.42	−2.7	[32]
Hydroxyproline	5.8–10.9	n.m.	5.94–5.58	−0.9	none
Leucine	155–254	74–125 35–66	143–301	+2.1	[33]
Lysine	110–171	115–223	96–169	+1.8	[33]
Methionine	41–59	17–30	35–55	+1.6	[33]
Ornithine	see Cit	25–68	18–96	+5.3	[33]
Phenylalanine	39–69	42–66	36–105	+2.9	[33]
Proline	118–230	70–188	68–86	+1.3	[33]
Sarcosine	1.3–2.6	n.m.	1.12–1.25	+1.1	[33]
SDMA	n.m.	n.m.	1.48–1.52	+1.4	[30]
Serine	106–210	80–157	52–175	+3.4	[33]
Threonine	105–162	68–169	53–101	+1.9	[33]
Tryptophan	6.3–20.4	41–68	5.7–34.3	+6.0	[33]
Tyrosine	36–64	40–74	31–58	+1.9	[33]
Valine	218–286	138–243	141–296	+2.1	[33]
N^ε^MK	n.m.	n.m.	1.14–1.19	+1.04	[27]

n.a., not applicable; n.m., not measured.

## Data Availability

Data are available on request.
